# Reactive oxygen species attenuation improves the quality of vitrified-warmed bovine embryos

**DOI:** 10.1590/1984-3143-AR2024-0035

**Published:** 2025-01-20

**Authors:** Viviane Luzia da Silva Feuchard, Clara Slade Oliveira, Naiara Zoccal Saraiva, Carolina Capobiango Romano Quintão, Leticia Zoccolaro Oliveira

**Affiliations:** 1 Departamento de Clínica e Cirurgia Veterinárias, Escola de Veterinária, Universidade Federal de Minas Gerais, Belo Horizonte, MG, Brasil; 2 Embrapa Gado de Leite, Juiz de Fora, MG, Brasil

**Keywords:** antioxidant, blastocyst, oxidative stress, vitrification

## Abstract

The aim of this study was to investigate the effects of modulating reactive oxygen species (ROS) in vitrified bovine in vitro produced (IVP) embryos. In experiment I we compared ROS production in fresh and vitrified-warmed blastocysts. In experiment II we evaluated the effects of antioxidant supplementation (100 μM of 2-mercaptoethanol; BME; 0 h to 2 h during warming) on ROS levels in vitrified-warmed blastocysts, and in experiment III we compared the development of fresh and vitrified-warmed blastocysts in the presence (BME) or absence (Control) of antioxidant (100 μM BME; 0 h to 48 h during warming). Higher ROS production (Fresh: 68.48 ± 7.92 *vs* Vitrified: 123.53 ± 13.15; P<0.05) and lower cell number was observed in vitrified compared to fresh embryos (Fresh: 123.01 ± 5.67 *vs* Vitrified: 103.04 ± 4.25; P<0.05). Antioxidant supplementation reduced ROS levels (Vitrified: 38.24 ± 1.27 vs. Vitrified/BME: 33.54 ± 1.08; P<0.05) and increased cell number in treated embryos (Vitrified: 100.65 ± 3.98 *vs*. Vitrified/BME: 112.95 ± 3.72; P<0.05). No differences were observed in the re-expansion rates of vitrified embryos cultured in the absence and presence of BME at 0, 2, and 4 h after warming (P>0.05). The embryo hatching rate did not differ (P>0.05) among embryos from the fresh, vitrified and vitrified/BME groups. However, the total cell numbers were higher (P<0.05) in vitrified embryos supplemented with BME (143.02 ± 6.97) than in vitrified embryos without BME (113.25 ± 5.09) but similar (P>0.05) to that observed in fresh embryos cultured with (150.54 ± 8.99) and without BME (142.71 ± 13.60). It was concluded that the vitrification and warming processes increased ROS levels in blastocysts and its attenuation with BME antioxidant improved embryonic quality.

## Introduction

Vitrification is the most used methodology to cryopreserve *in vitro* produced embryos from different species (González-Rodríguez et al., 2022; Fryc et al., 2023; Huang et al., 2024) due to better embryonic survival rates after warming process when compared to slow freezing (Caamaño et al., 2015; Martínez-Rodero et al., 2022).

However, despite achieving a 95% survival rate for IVF embryos and respectable pregnancy rates in bovines through cryopreservation and direct transfer (Oliveira et al., 2020), we recognize the need for further enhancing the quality of cryopreserved embryos.

Vitrification has been associated with increased reactive oxygen species (ROS) levels in murine (Martino et al., 2013), swine (Xiang et al., 2022), and bovine (Fabra et al., 2023) embryos, in addition to a reduction in antioxidant biomarker levels (total antioxidant capacity, superoxide dismutase and glutathione peroxidase) (Alshaheen et al., 2021) and intracellular concentrations of glutathione (GSH) (Gao et al., 2012; Nohalez et al., 2018), a thiol tripeptide which is responsible for cellular protection against oxidative damage (Battin and Brumaghim, 2009).

High intracellular levels of ROS activate the apoptosis cascade (Orrenius et al., 2015; Paschoal et al., 2017; Moussa et al., 2019) and apoptotic biomarkers (Bcl-2 associated X protein, heat shock 60kD protein member 1, tumor necrosis factor alpha) have been described in vitrified embryos (Alshaheen et al., 2021), contributing to the reduction of its quality and capacity for development after cryopreservation (Valente et al., 2020). Thus, vitrification-induced oxidative stress can affect the development and quality of cryopreserved embryos (Mittler et al., 2011; López-Damián et al., 2020; Soto-Heras and Paramio, 2020).

Therefore, using an experimental strategy of culturing vitrified embryos after the warming stage in the presence of 2-mercaptoethanol (BME), a well-characterized antioxidant for bovine embryos (De Matos and Furnus, 2000; Takahashi et al., 2002), we investigated the effects of modulating reactive oxygen species in vitrified bovine blastocysts, based on the premise that oxidative stress plays a relevant role in vitrified embryo quality.

## Methods

This study followed research ethical guidelines and do not require a CEUA protocol since was no live animals were included in the study.

### Chemical reagents

All chemicals used in the experiments were obtained from Sigma-Aldrich (Merck, Darmstadt, Germany) unless otherwise specified.

### Experimental design

Initially (Experiment I, three replicates), fresh and vitrified-warmed blastocysts (D7) were cultured for 2 h without antioxidant. Then, they were stained with the CellRox Green and Hoechst 33342 fluorogenic probes to investigate the influence of vitrification/warming process on ROS levels and whether they could promote changes concerning the total number of cells in vitrified embryos.

In experiment II (three replicates), the investigation focused on whether, after warming the embryos, the use of the antioxidant 2-mercaptoethanol (BME; 100 μM) could neutralize ROS production, reducing their levels and improving embryo quality by increasing the total number of cells. This concentration was utilized given its association with increased intracellular concentrations of glutathione (Takahashi et al., 1993) and was adopted for our study based on related literature (De Matos and Furnus, 2000; Nedambale et al., 2006; De Mattos et al., 2022). Vitrified/warmed embryos were segregated into two experimental groups: embryos cultured in the absence (Vitrified) and in the presence (Vitrified-BME) of antioxidant and cultured for 2 h. Blastocysts were then stained with CellRox Green and Hoechst 33342 to determine oxidative stress and total cell number.

Subsequently, in Experiment III (three replicates) we investigated the effects of antioxidant supplementation on embryonic development within 48 h after warming. Fresh and vitrified-warmed embryos were cultured in the presence (100 μM) (Fresh-BME; Vitrified-BME) and absence of BME (Fresh; Vitrified) and each experimental group was evaluated for re-expansion, hatching, oxidative stress and total number of cells.

### *In vitro* embryo production

All culture steps (IVM, IVF, IVC and vitrified embryo warming) were performed at 38.5°C, 5% CO_2_ in atmospheric air, and high humidity under a layer of mineral oil.

Bovine ovaries were collected in a local slaughterhouse and transported to the laboratory in saline solution at 37ºC. The interval from ovary collection to its processing amounted to approximately 4 hours. The recovery of cumulus-oocyte complexes (COCs) was performed by aspirating follicles of 2 to 6 mm in diameter. The experiment only used COCs presenting homogeneous cytoplasm with two or more layers of cumulus cells. After selection, COCs were matured in groups of 25, for 24 h, in 100 µl drops of MIV medium (TCM-199 supplemented with 10% FCS, 1 μg/mL FSH, 50 μg/mL hCG, 1 μg/mL 17β estradiol, 16 μg/mL sodium pyruvate, 10,000 IU penicillin and 10 mg streptomycin/mL). After MIV, COCs were transferred in groups of 20 to 100 μl drops of Fert-Talp medium (supplemented with 0.6% BSA, 10 μg/mL heparin, 18 μM penicillamine, 10 μM hypotaurine, and 1.8 μM epinephrine). After thawing the semen (previously tested from a single bull), the motile sperm were selected in a Percoll discontinuous gradient (45%/90%) and subsequently added to the fertilization drops at a concentration of 2 x 10^6^ motile sperm/mL. IVF was performed for 18 h. Probable zygotes were denuded, washed and transferred in groups of 20 structures to drops of 70µl of Synthetic oviductal fluid with amino acids (SOF-AA) (supplemented with 1.5% FCS and 6 mg/mL BSA medium). *In vitro* culture was performed for 7 d and 50% of the media was exchanged on D3 and D6.

### Vitrification and warming

Grade 1 blastocysts (according to IETS classification; Stringfellow and Givens, 2010) were selected and vitrified on D7 using a two-step protocol.

Vitrification was performed in groups of five embryos. Holding media was used for all vitrification-warming procedures (TCM-199 buffered with HEPES (Gibco BRL, Grand Island, NY) supplemented with 1.0 mM sodium pyruvate (Gibco BRL, Grand Island, NY), 100 UI penicillin and 0.1 mg/ml streptomycin. For vitrification, holding media was supplemented with 10% fetal calf serum (FCS). Selected embryos were transferred to 200 μl 7.5% DMSO +7.5% ethylenoglycol media for 3 min. Next, embryos were washed in 200 μl 16% DMSO +16% ethylenoglycol +0.5 M sucrose media and placed in an open vitrification device (WTA, Cravinhos, Brazil) in a 0.5 μl droplet. After 30 s device was immersed in liquid nitrogen.

For warming, holding media was supplemented with 1% FCS and was perfomed as previously described (Oliveira et al., 2020) by placing vitrification device directly from liquid nitrogen into 0.15 M sucrose media colum, where embryos remained for 6 min. After warming, they were washed three times in 1% FCS PBS and then transferred to drops containing a SOF-AA medium.

### 2-mercaptoethanol solution

A 55 mM 2-mercaptoethanol stock solution (Gibco BRL, Grand Island, NY) was diluted in phosphate-buffered saline to prepare a 1 mM solution and stored at 4ºC until use.

### Post-warming culture

In experiments I and II, embryos were cultured for 2 h in groups of 20 structures in 100 μL drops of SOF-AA medium. In Experiment II, embryos in the treated group (vitrified BME) were cultured with the antioxidant from the beginning until the end of the culture period (0 h to 2 h). One hour after beginning the culture, embryos from each experimental group were stained, still in the drops, for 1 h with CellRox Green and Hoechst to evaluate the oxidative stress and total number of cells.

In Experiment III, embryos in the treated groups (fresh BME and vitrified BME) were cultured with BME from the beginning to the end of the culture period (0 h to 48 h). During the culture period, vitrified-warmed embryos (Vitrified Control and Vitrified BME) were evaluated for re-expansion at 0, 2, and 4 h after warming. At the end of the culture, embryos were evaluated for hatching, oxidative stress, and total cell number.

### Re-expansion

The re-expansion rate of vitrified-warmed embryos was performed by evaluating the images of each embryo at 0, 2, and 4 h after warming. Re-expansion was considered positive when the embryo’s blastocele was observed in the image.

### Oxidative stress and total number of cells

At this stage, the embryos were incubated for 1 hour in SOF-AA medium at 38.5°C and high humidity, under a layer of mineral oil. To determine oxidative stress and total cell number, the embryos were stained with the fluorescent probes CellRox Green (Invitrogen Molecular Probes, USA) and Hoechst 33342 (10 µg/mL). The Hoechst stock solution was diluted in phosphate-buffered saline (1000 µg/mL; 100x). Both CellRox Green and Hoechst stock solutions were stored frozen. Subsequently, the fluorescent dyes were added simultaneously to the droplets containing the embryos (1:100). Throughout the staining period, the embryos were kept in a gas incubator (5% CO2 in atmospheric air), shielded from light.

Blastocysts were stained for 1 h, washed in TCM-199 after incubation, and individually imaged using a fluorescence microscope (EVOS M5000, Thermo Fisher Scientific, USA) with 357 nm excitation and 525 nm emission. Images of the fluorescent blastocysts were analyzed with ImageJ Software (National Institutes of Health, Maryland, USA) to measure pixels in the blastocyst area and to determine the total number of cells.

Fluorescence intensity was calculated by averaging pixels after manually selecting the area of each embryo. The area selected to measure the fluorescence intensity was the entire embryonic area present within the zona pellucida. The total number of cells was determined by manually counting the nuclei of each blastocyst and the oxidative stress of each embryo was calculated by dividing the ROS levels by the total number of blastomeres.

### Hatching

Hatching was evaluated at 48 h post-warming by evaluating the images of each embryo. The hatching rate was calculated based on the number of re-expanded embryos. Blastocysts were considered hatched if they presented a ruptured zona pellucida and external projection of the embryonic portion.

### Statistical analysis

In Experiment I, oxidative stress was compared using the Mann-Whitney test, while the total cell count was assessed using the T-test. Experiment II compared oxidative stress and total cell count using the T-test. Experiment III evaluated re-expansion and hatching means with Fisher's Exact Test, oxidative stress with the Kruskal-Wallis test, and total cell count Kruskal Wallis and Dunn post Test. A significance level of 5% was adopted, and analyses were performed using Minitab Software (version 21.4.1.0).

## Results

### Vitrification and warming alter the oxidative stress and total cell number of cryopreserved embryos

**Figure 1:**
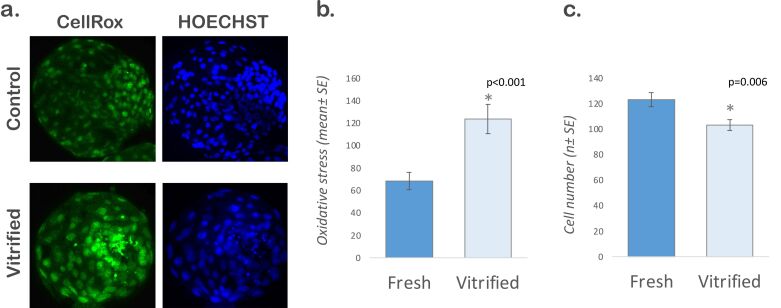
Levels of reactive oxygen species and total cell count in fresh and vitrified embryos 2 h after warming. a. Fresh and vitrified-warmed blastocysts stained with CellRox and HOECHST 33342. b. Graph for oxidative stress. c. Graph for total cell count. The quantitative data are presented as mean ± standard error. Asterisks (*) indicate statistical difference (P<0.001). For this experiment, 133 blastocysts obtained in 3 replicates were analyzed (61-72 per group).

To evaluate the influence of vitrification/warming on the production of reactive oxygen species in cryopreserved embryos, the oxidative stress and total number of cells in fresh and vitrified-warmed embryos (*n* = 133 blastocysts obtained in three replicates, 61-72 per group) were determined in experiment I. An increase in the oxidative stress (68.48 ± 7.92 *vs*. 123.53 ± 13.15; *P*<0.001) and a reduction in the total number of cells (123.01 ± 5.67 *vs*. 103.04 ± 4.25; *P*=0.006) were observed in vitrified-warmed embryos (Figure 1).

### Attenuation of oxidative stress reduces the oxidative stress and increases the total cell number of vitrified-warmed embryos

**Figure 2:**
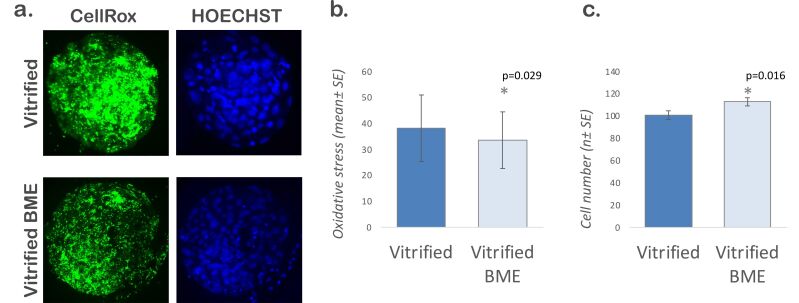
Effects of post-warming culture with 2-mercaptoethanol. a. Image of vitrified-warmed blastocysts cultured without and with beta-mercaptoethanol stained with CellRox and HOECHST 33342 2 h after warming. b. Graph for oxidative stress. c. Graph for total number of cells. The quantitative data are presented as mean ± standard error. Asterisks (*) indicate statistical difference (P<0.05). For this experiment, 136 blastocysts obtained in 3 replicates were analyzed (55-81 per group).

The experiment II investigated whether supplementation with BME could reduce oxidative stress and increase the total number of cells in vitrified embryos (*n* = 136 blastocysts obtained in three replicates, 55-81 per group). Our experimental conditions revealed that the culture of vitrified embryos warmed in the presence of BME reduced the oxidative stress (38.24 ± 1.27 *vs*. 33.54 ± 1.08; *P*<0.05) and increased the total number of cells (100.65 ± 3.98 *vs*. 112.95 ± 3.72; *P*<0.05) (Figure 2).

### Reduction of the post-warming oxidative stress with 2-mercaptoethanol does not alter cryosurvival in the short term but increases the total number of cells in vitrified embryos

**Figure 3:**
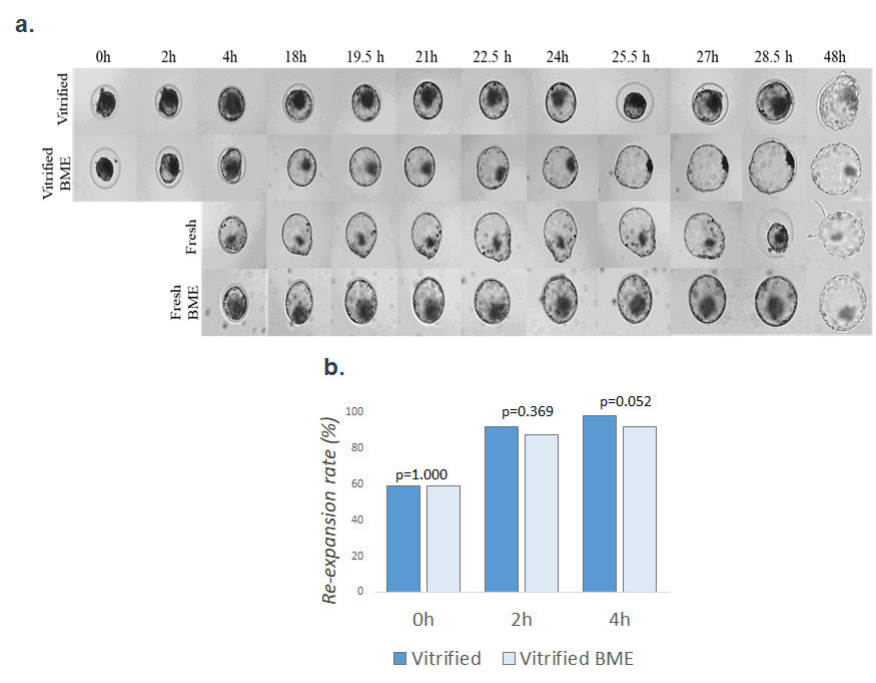
Effects of post-warming culture with 2-mercaptoethanol on the development of vitrified embryos. a) Image shows embryonic development of fresh and vitrified embryos cultured without and with beta-mercaptoethanol. b) Graph shows rate of embryonic re-expansion at 0, 2 and 4 h after warming. For this experiment, 212 blastocysts obtained in 3 replicates were analyzed (109-103 per group).

The re-expansion rates of vitrified embryos cultured in the absence and presence of BME (*n* = 212 blastocysts obtained in three replicates, 109-103 per group, respectively) were evaluated at 0, 2, and 4 h after warming during experiment III. No differences were detected (*P*>0.05) between the groups at 0 h (58.72% *vs*. 59,22%), 2 h (91,74% *vs*. 87,38%) and 4 h (98.17% *vs*. 92.08%) (Figure 3).

**Figure 4:**
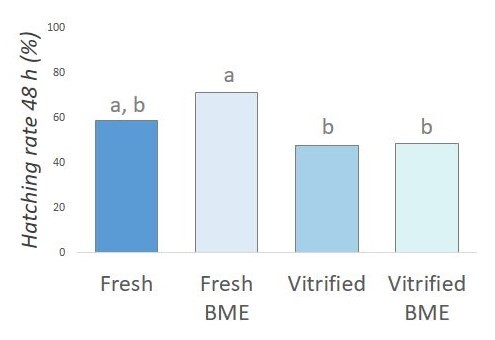
Cryosurvival of fresh and vitrified embryos after post-warming culture with 2-mercaptoethanol. Hatching rate of fresh and vitrified embryos cultured in the presence and absence of BME after 48 h of culture. For this experiment, 241 blastocysts obtained in 3 replicates were analyzed (39-38-84-80 per group). Different letters indicate means are not equal (P<0.05).

Hatching rates (*n* = 241 blastocysts obtained in three replicates, 39-38-84-80) did not differ (*P*>0.05) among embryos from the fresh (58.97%), vitrified (47.62%) and vitrified-BME (48.75%) groups (Figure 4).

**Figure 5:**
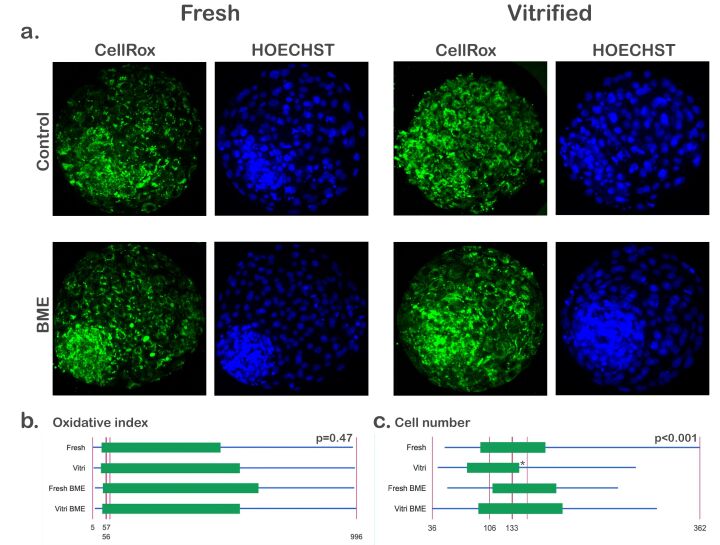
Total cell number and oxidative stress levels of fresh and vitrified embryos cultured with 2-mercaptoethanol (BME) after 48 h of culture. a. Images of stained embryos stained with CellRox and HOECHST 33342. b. Boxplot for oxidative stress. c. Boxplot for total cell number. Asterisk (*) denotes means are not equal. For the oxidative stress experiment, 222 blastocysts obtained in 3 replicates were analyzed (27-74-39-82 per group). For the total cell count, 243 blastocysts obtained in 3 replicates were analyzed (28-91-39-85 per group).

The oxidative stress of blastocysts (*n* = 222 blastocysts obtained in three replicates; 27-74-39-82, per group) did not differ (*P*>0.05) among the fresh, vitrified, fresh BME and vitrified-BME groups (287.49 ± 26.27, 368.95 ± 24.97, 317.07 ± 25.42, 363.85 ± 25.35, respectively) (Figure 5).

The total number of cells (*n* = 243 blastocysts obtained in three replicates, 28-91-39-85 per group) in vitrified embryos cultured with BME (143.02 ± 6.97) was higher (*P*<0.05) than in vitrified embryos without BME (113.25 ± 5.09) but similar (*P*>0.05) to that observed in fresh embryos cultured with (150.54 ± 8.99) and without BME (142.71 ± 13.60) (Figure 5).

## Discussion

Based on the hypothesis that the production of free radicals at the time of warming negatively affects the survival and quality of vitrified embryos, this study aimed to understand the relationship between ROS levels and post-warming embryonic survival. Hence, we initially investigated whether the vitrification/warming process could alter intracellular ROS levels and the total number of cells in vitrified blastocysts (Experiment I). Then, we evaluated the effects of BME supplementation on ROS production and total cell number in these embryos (Experiment II) and, further, this antioxidant was subsequently used in post-warming culture to neutralize these free radicals and verify possible improvements in the viability and quality of vitrified embryos (Experiment III). Our results shed light on the participation of oxidative stress in the survival of vitrified bovine embryos, highlighting the importance of its modulation.

The findings revealed that vitrification/warming increased ROS levels which has been previously observed in vitrified mouse (Martino et al., 2013) and porcine (Nohalez et al., 2018) embryos. Moreover, previous studies recently demonstrated that slow freezing (Lopez-Damian et al., 2020) and vitrification (Silva et al., 2021) of bovine embryos were associated with an increase in ROS production, indicating that the development of oxidative stress was favored by the cryopreservation process increasing the proportion of free radicals and/or reducing the concentration of antioxidants.

Vitrification has been reported to cause mitochondrial dysfunction (Dalcin et al., 2013; Hara et al., 2018), which leads to an imbalance between the production and removal of the ROS molecules (Lin and Beal, 2006). Elevated levels of ROS can trigger lipid peroxidation (Maia et al., 2010) and mitochondrial DNA damage which reduces mitochondrial membrane potential and ATP synthesis (Orrenius et al., 2015), resulting in diminished vitrified embryo development capacity (Hara et al., 2018) and/or reducing the total cell number in cryopreserved embryos (Silva et al., 2021). Additionally, in porcine blastocysts, vitrification and warming increased ROS production and reduced the glutathione levels, a non-enzymatic antioxidant (Nohalez et al., 2018).

Embryos are exposed to a non-physiological microenvironment during *in vitro* culture, one that lacks antioxidant substances, which would be available during *in vivo* development (Wang et al., 2002; Jamil et al., 2020). They are also exposed to pro-oxidant factors (composition of the culture medium, cryopreservation, pH, oxygen tension temperature) that contribute to the increase in ROS production (Soto-Heras and Paramio, 2020; Agarwal et al., 2022). It is known that embryo culture under higher oxygen tension (20%) is associated with increased ROS levels in embryos (Amin et al., 2014; Leite et al., 2018), due to metabolic alterations (increased pyruvate consumption) and heightened mitochondrial activity (De Lima et al., 2020). Elevated ROS levels may overwhelm embryo defense mechanisms (Sciorio and Smith, 2019) and consequently alter the expression of antioxidant genes (Ma et al., 2017).

Thus, the antioxidant 2-mercaptoethanol has been used during oocyte maturation (Patel et al., 2015) and embryo culture (De Mattos et al., 2022) to neutralize ROS levels by inducing the synthesis of intracellular glutathione, which occurs by reducing cystine to cysteine (Issels et al., 1988). It was already demonstrated that a 100 μM of BME increased the intracellular concentration of glutathione (Takahashi et al., 1993) and when added to the culture medium for warmed embryos, it was shown to enhance both the survival and quality of embryos post-vitrification (Nedambale et al., 2006).

The results of Experiment II demonstrated that supplementation of 100 μM BME in the post-vitrification culture medium reduced ROS levels and increased the total number of cells in vitrified embryos. The increase in the total number of cells in vitrified embryos after cultivation in the presence of this antioxidant has also been observed in other studies (Nedambale et al., 2006; De Mattos et al., 2022), corroborating our finding.

Although we did not observe an increase in re-expansion and hatching rates at the evaluated times (Figures 3 and 4), we found that BME treatment was able to promote an increase in the total number of cells in vitrified embryos when compared to the control vitrified group, suggesting that oxidative stress attenuation improves embryonic quality (Figure 5).

Still, the results regarding to the effects of the antioxidant employed on embryo survival were similar to described by other authors (Rocha-Frigoni et al., 2014; De Mattos et al., 2022). Moreover, worth mentioning that the present study used a column warming technique with a single solution, which improves embryonic survival and quality (Oliveira et al., 2020). It is perhaps for that reason that, regardless of the presence of BME, the hatching rate of vitrified embryos remained similar to that observed in fresh embryos (Figure 4), making it difficult to perceive the effects of this antioxidant on embryonic hatching. Furthermore, the findings did not reveal a significant reduction in ROS levels at the end of culture in vitrified embryos cultured with BME (Figure 5), which was also observed by other authors (Rocha-Frigoni et al., 2014). Thus, it is plausible that the vitrified blastocysts may have already reached intracellular redox equilibrium at the time of evaluation, resulting in similar levels of ROS between fresh and vitrified embryos after 48 hours of culture.

Fabra et al. (2023) demonstrated that the inclusion of alpha-lipoic acid (ALA), a coenzyme that plays a key role in mitochondrial multienzyme complex reactions in charge of recycling other cellular antioxidants, such as a glutathione content, in the *in vitro* culture medium presented positive effects on embryo development and cryotolerance after vitrification of bovine embryos. Still, the authors also demonstrated that, although supplementation with ALA increased blastocyst total cell number and the percentage of excellent-quality embryos, the inclusion of ALA did not modify viability and ROS levels evaluated after an acute treatment (3 h) in zygotes, and at the end of 24 h of treatment in day 2 cleaved embryos. This would indicate that despite the effect of antioxidant was not quickly evidenced, it was key to embryonic development (Fabra et al., 2023).

Although at physiological levels ROS play an important role in cell signaling (Mittler et al., 2011), high levels of ROS are responsible for the activation of mitogen-activated protein kinases (JNK and p38) (Guyton et al., 1996; Dabrowski et al., 2000), DNA damage, and apoptosis (López-Damián et al., 2020; Kang et al., 2021). A recent study demonstrated that embryos submitted to oxidative stress conditions had a concomitant increase in JNK phosphorylation and apoptosis (Zhuang et al., 2020), whereas reduced levels of these free radicals were related to reduced protein kinase and apoptosis levels (Yu et al., 2021). Hence, the use of BME may have inhibited the activation of JNK and P38 pathways, while signaling pathways involved in cell proliferation - such as protein kinase B (Akt) - may have been activated due to reduced intracellular levels of ROS, reducing cell death, and increasing the total number of cells in cryopreserved embryos. However, other studies are necessary to elucidate the relationship between the use of BME and the activation of specific pathways related to cell proliferation and death.

## Conclusion

We concluded that, under the conditions provided, the vitrification and warming process influenced the increase in ROS production in bovine blastocysts. Although reducing the levels of these free radicals, supplementation with 100 μM of 2-mercaptoethanol did not affect survival during early development but improved the total cell number of vitrified embryos, signaling a possible benefit of modulating oxidative stress on the quality of embryos cryopreserved by vitrification.
